# Expression and cell transformation activity of dynactin‐associated protein isoforms

**DOI:** 10.1002/2211-5463.13202

**Published:** 2021-07-16

**Authors:** Xiaobo Yin, Shota Yamada, Hiroaki Kobayashi, Ryota Tanaka, Yuki Togo, Miho Hosoi, Mie Tsuchida, Tatsuki Kunoh, Shuichi Wada, Toshinobu Nakamura, Ryuzo Sasaki, Tamio Mizukami, Makoto Hasegawa

**Affiliations:** ^1^ Faculty of Bioscience Nagahama Institute of Bio‐Science and Technology Japan; ^2^ Frontier Pharma Nagahama Japan

**Keywords:** alternative splicing, cell transformation, dynactin‐associated protein, spheroid formation, subcellular localization

## Abstract

Overexpression of human dynactin‐associated protein isoform a (dynAPa) transforms NIH3T3 cells. DynAPa is a single‐pass transmembrane protein with a carboxy‐terminal region exposed to the outside of cells. According to the NCBI RefSeq database, there may be two other splicing variants of the encoding gene (dynAPb and c). DynAPa and c differ in some amino‐terminal residues (NH_2_‐MVA in dynAPa and NH_2_‐MEYQLL in dynAPc). DynAPb has the same amino‐terminal residues as dynAPc, but lacks 55 residues in the intracellular region. All three isoforms have the same carboxy‐terminal region, including the transmembrane domain. Expression of mRNAs of three splicing variants was found in human cancer cell lines ACHN and Caki‐1. The subcellular localization and *in vitro* cell transformation ability of the three isoforms were examined using NIH3T3 cells overexpressing each respective isoform. All isoforms were found to be localized to the Golgi apparatus and plasma membrane, where the carboxy‐terminal region was exposed to the outside of cells. Cell transformation was tested using focus formation due to loss of contact inhibition of cell proliferation, and colony formation was examined on soft agar and spheroid formation in ultralow U‐bottomed wells. DynAPa robustly formed foci and colonies on soft agar and spheroid, whereas these abilities were considerably decreased for dynAPb and completely lost in dynAPc. These findings warrant dissection studies to identify the dynAP domain that is required for cell transformation.

## Graphical abstract

Human dynactin‐related protein dynAP and its splicing variants were found to have different amino‐terminal residues or to lack residues in the intracellular region. Spheroid formation showed that dynAPa has the highest cell transformation activity, which was significantly diminished in dynAPb and completely lost in dynAPc. The results will assist future identification of the amino acid residues required for cell transformation activity.

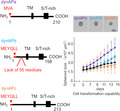

**Abbreviations**
CCT: chaperonin‐containing tailless complex polypeptide 1
DAPI: 4′,6‐diamidino‐2‐phenylindole
DMEM: Dulbecco's modified Eagle's medium
dynAP: dynactin‐associated protein
FACS: fluorescence‐activated cell sorting
FCS: fetal calf serum
TM: transmembrane

Interaction between dynactin and a dynein motor protein is required for microtubule‐mediated movement and spatial organization of intracellular vesicles, organelles, and macromolecular complexes [1, 2, 3]. Dynactin is a large protein complex including components that bind various cargos, dynein, and microtubules. Interaction between dynactin and dynein also plays critical roles in positioning the mitotic spindle during cell division [4, 5]. A previous study on human proteins that might be involved in cell proliferation events led us to discover the C18orf26 protein, which we named dynactin‐associated protein (dynAP) because it physically interacts with dynactin components such as dynamitin and p150^Glued^ [6].

Chaperonin‐containing tailless complex polypeptide 1 (CCT), which consists of eight distinct subunits (CCTα~θ), functions as a molecular chaperone for the folding of tubulin and actin, and thereby controls the composition of the cytoskeleton, including microtubules [7]. In addition to performing a critical role as a subunit of the CCT complex, monomeric CCTδ interacts with p150^Glued^, and this interaction is required for the formation of cell surface protrusions. Depletion of dynAP or p150^Glued^ prevents the localization of monomeric CCTδ to the plasma membrane, suggesting that association of CCTδ with p150^Glued^ and dynAP close to the plasma membrane contributes to increasing cell movement [8]. Dynein and dynactin complex are both localized to the leading edge of cells and contribute to cell orientation and persistent cell migration. Since wound healing assays showed that NIH3T3 cells overexpressing dynAP move faster than control cells [9], the presence of dynAP in close proximity to the plasma membrane with CCTδ bound to p150^Glued^ may contribute to increasing directional cell migration.

DynAP is a type II single‐pass transmembrane (TM) protein consisting of 210 amino acids with its C‐terminal region exposed on the outside of cells. Human dynAP transforms NIH3T3 cells [9], and NIH3T3dynAP cells (NIH3T3 cells overexpressing human dynAP) display the hallmarks of *in vitro* cell transformation, such as foci formation in 2D culture, colonies on soft agar, and spheroids in 3D culture. Furthermore, injection of NIH3T3dynAP cells into nude mice results in tumors with abundant blood vessels and weak cell–cell contacts.

Several human cancer cell lines express dynAP [6], but expression in normal human tissues is limited in esophagus and spleen [10], and its physiological function remains to be determined. According to the National Center for Biotechnology Information (NCBI) RefSeq database, there are three possible splicing variants of the human dynAP gene. We explored the dynAPa isoform in previous work. Since it was established that the splicing pattern of many genes is closely related to tumorigenesis, cancer metastasis, and drug resistance (reviewed in Refs [11, 12, 13, 14]), it is worth investigating the other dynAP isoforms. In the present work, we separately overexpressed each of the dynAPa–c isoforms in NIH3T3 cells and analyzed their subcellular localization and *in vitro* cell transformation capability.

## Materials and methods

### Cell culture

Mouse NIH3T3‐3‐4 cells (subcloned from NIH3T3 cells) were maintained in Dulbecco's modified Eagle's medium (DMEM) supplemented with 4.5 g·L^−1^ glucose (Nacalai Tesque, Inc., Kyoto, Japan), 100 U·mL^−1^ penicillin, 100 μg·mL^−1^ streptomycin (Nacalai Tesque, Inc.), and 10% fetal calf serum (FCS; SAFC Biosciences, Inc., Lenexa, KS, USA). Hereafter, this cell line is referred to as NIH3T3. Human ACHN, Caki‐1, MCF‐7, PC‐3, HeLa, DLD‐1, and KMST‐6 cells were maintained in RPMI1640 medium (Sigma‐Aldrich Co. LLC, St. Louis, MO, USA) supplemented with 10% (v/v) FBS (SAFC Biosciences, Inc.), 100 μg·mL^−1^ streptomycin, and 100 U·mL^−1^ penicillin. Cells were cultured in a CO_2_ incubator at 37 °C with 5% CO_2_.

### Reverse transcription‐PCR

Total RNA was isolated using an RNeasy Mini kit (Qiagen N.V., North Rhine‐ Westphalia, Germany) according to the manufacturer's protocol. Reverse transcription‐PCR was conducted with ReverTraAce‐α (Toyobo Co., Ltd., Osaka, Japan) using 1 μg of each total RNA and primers amplifying dynAPa–c cDNAs as follows: #0, 5′‐tcacccaaaaatggaataccaacttctag‐3′; #1, 5′‐gaagatctagttgcagatatataaagggcaat‐3′; #2, 5′‐acgcgtcgacttataaatgatcggtaggtg‐3′; #10, 5′‐ctgtgtgttacaggattctgactgtag‐3′. As an internal control, the mRNA of glyceraldehyde‐3‐phosphate dehydrogenase was amplified using primers 5′‐accacagtccatgccatcac‐3′ and 5′‐tccaccaccctgttgctgta‐3′.

### Antibodies against dynAP

**Fig. 1:**
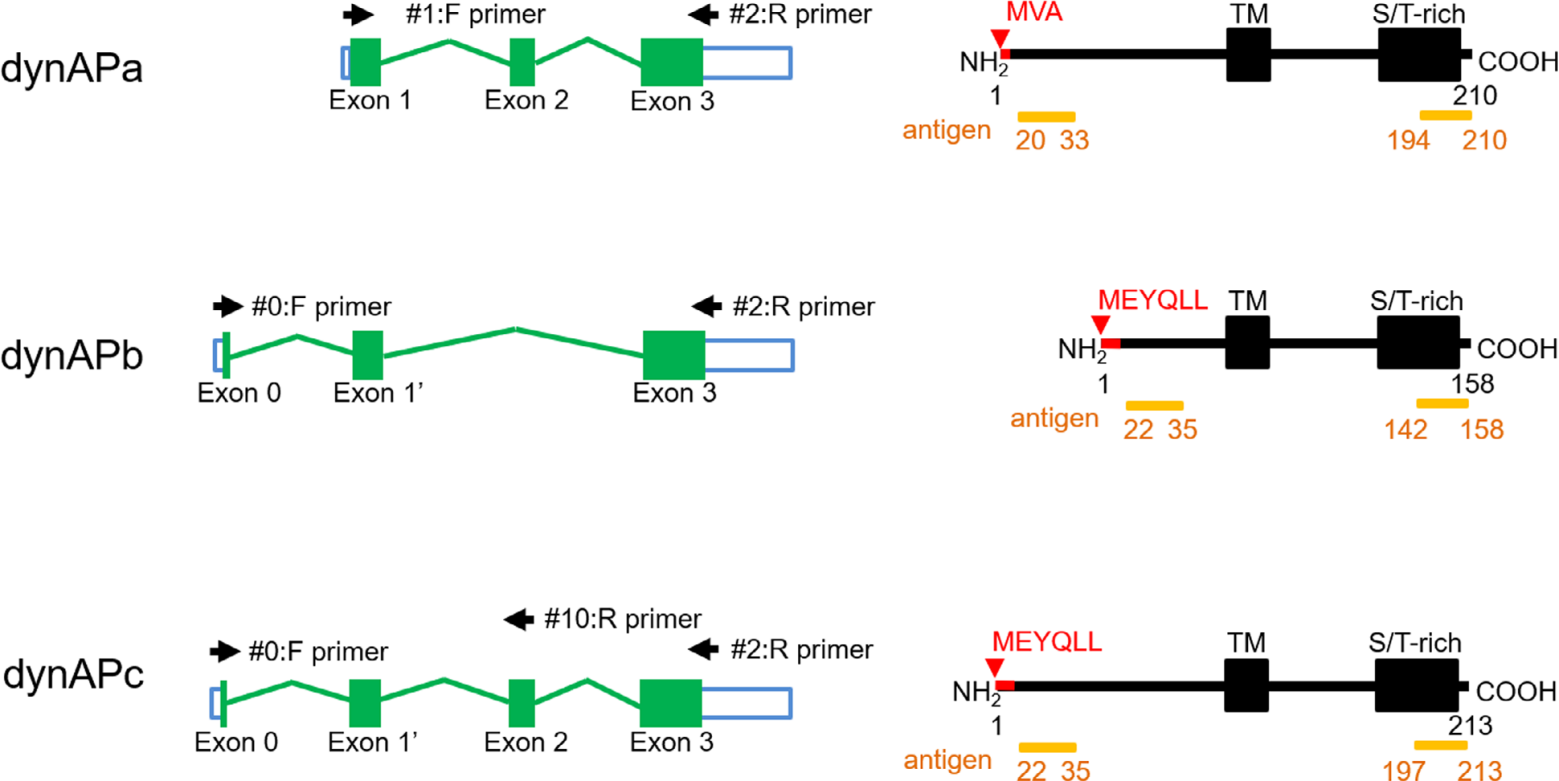
Isoforms of dynAPa, b, and c. Green boxes in exons represent protein‐coding regions, and white boxes represent noncoding regions. Green lines show introns. Numbers indicate amino acid residues. TM, transmembrane region. Amino acid residues around the N terminus are specified for comparison. The primary transcript of variant dynAPa contains three exons (exon 1–3). The primary transcript of variant dynAPb contains exons 0, 1′, and 3, but lacks exon 2. Exon 0 contains 19 bases encoding 6 + 1/3 amino acids, and exon 1′ contains 125 bases encoding 2/3 + 41 amino acids; hence, exon 0 and exon 1′ together encode 48 amino acids. The primary transcript of valiant dynAPc contains exons 0, 1′, 2, and 3. Exon 3 is present in all mRNAs encoding the TM region and the S/T‐rich C‐terminal region. DynAPb and c differ from dynAPa in amino acid sequences within the N‐terminal region. Antigens (residues 23–33 and 194–210) in dynAPa indicate amino acid sequences used to raise N‐ and C‐antibodies, respectively. Since these sequences are present in both dynAPb and c, these antibodies recognize all three isoforms. Arrows indicate primers used to amplify mRNA‐derived DNA fragments, and F and R refer to forward and reverse directions.

Two polyclonal antibodies that can recognize all dynAP isoforms were raised by immunizing rabbits with N‐antigen (residues 20–33) and C‐antigen (residues 194–210) in dynAPa (Fig. 1 and Fig. S1). N‐ and C‐antibody indicate IgG fractions prepared from sera of rabbits receiving C‐antigen, respectively. IgG was purified from rabbit sera by affinity chromatography against antigen peptides. In a previous study, the N‐antibody (referred as anti‐dynAP antibody [9]) was shown to detect specifically human dynAP (dynAPa in this paper) in NIH3T3dynAP cells that overexpress dynAP [9]. Control IgG from non‐immunized rabbits was purchased from Wako Pure Chemical Inc., (Osaka, Japan).

### Expression of dynAP isoforms

Full‐length cDNAs encoding dynAPa (NCBI accession number: NM_173629.1), dynAPb (NCBI accession number: NM_001307955.1), and dynAPc (NCBI accession number: XM_011525924.2) were subcloned into the pFLAG‐CMV2‐Bsd vector, a derivative of pFLAG‐CMV‐2 (Sigma‐Aldrich) carrying the blasticidin S deaminase gene, yielding plasmids expressing N‐terminally Flag‐tagged dynAPa–c. For retrovirus‐mediated expression, untagged dynAPa–c cDNAs were incorporated into the EcoRI and XhoI sites of the pMY‐IRES‐EGFP vector as described previously [9]. The resulting plasmids were introduced into Plat‐E cells using FuGENE 6 transfection reagent (Promega Co., Madison, WI, USA) according to the manufacturer's instructions. After 48 h, virus‐containing supernatants were filtered through 0.45 μm cellulose acetate filters and supplemented with 8 μg·mL^−1^ polybrene (Sigma‐Aldrich). NIH3T3 cells were then incubated overnight with the virus/polybrene‐containing supernatants. After infection, the medium was replaced with fresh medium. The expression, subcellular localization, and *in vitro* cell transformation ability were assessed in the resultant cells. Plasmids expressing N‐terminally Flag‐tagged dynAPa–c were transiently transfected into KMST‐6 cells using FuGENE HD (Promega), and subcellular localization of dynAPa–c was analyzed.

### Analysis of dynAP isoforms by SDS/PAGE

Lysates from NIH3T3 cells expressing dynAP isoforms were subjected to 12% SDS/PAGE. For western blotting, N‐antibody was used as the primary antibody, and goat anti‐rabbit IgG conjugated to horseradish peroxidase served as the secondary antibody.

### Subcellular localization

Cells were cultured on poly‐l‐lysine‐coated coverslips for 24 h and examined by indirect immunofluorescence microscopy. Briefly, cells were fixed in 70% methanol for 30 min and incubated in Blocking One (Nacalai Tesque) for 30 min. Cells were then incubated with antibodies against dynAP, FLAG (Medical & Biological Laboratories Co., Ltd., Aichi, Japan), or the Golgi marker GM130 (BD Transduction Laboratories, Becton, Dickinson and Company, Franklin Lakes, NJ, USA), followed by incubation with secondary antibodies conjugated with CF555 (Biotium Inc., Fremont, CA, USA) and Alexa Fluor 488 (Thermo Fisher Scientific, Waltham, MA, USA). After adding one drop of Fluoro‐KEEPER Antifade Reagent (Non‐Hardening Type) with 4′,6‐diamidino‐2‐phenylindole (DAPI; Nacalai Tesque), cell images were acquired by an Axioskop 2 plus microscope (Carl Zeiss AG, Thuringia, Germany). Line‐scan profiles were generated from each cell image using the plot profile function in the imagej/fiji (National Institutes of Health, Bethesda, MD, USA) image analysis platform.

### Flow cytometry

After gentle trypsinization, 3 × 10^5^ cells were resuspended in fluorescence‐activated cell sorting (FACS) buffer (2% FBS in phosphate‐buffered saline) and incubated with C‐antibody or nonspecific rabbit IgG (Wako Pure Chemical, Inc.), followed by incubation with secondary antibody R‐PE (Goat anti‐rabbit IgG R‐Phycoerythrin; SouthernBiotech Associates, Inc., Birmingham, AL, USA). After resuspension in FACS buffer containing 1 μg·mL^−1^ propidium iodide, cells were analyzed using an SH800 cell sorter (SONY, Tokyo, Japan).

### Formation of foci

Cells were seeded in a 6‐well tissue culture plate at a density of 10^4^ cells/well and cultured for 15 days. Cells were then fixed in ice‐cold methanol and stained with a 0.5% Crystal Violet (Nacalai Tesque) to identify the presence of cell colonies.

### Colony formation on soft agar

Soft agar assays were performed in 6‐cm dishes (Techno Plastic Products AG, Schaffhausen, Switzerland) containing cell growth medium (DMEM plus 10% FCS) supplemented with 100 U·mL^−1^ penicillin and 100 μg·mL^−1^ streptomycin. Experiments were performed in triplicate. For each dish, 2 × 10^4^ cells were mixed thoroughly with 0.3% agar (#01028‐85; Nacalai Tesque); the mixture (2 mL) was plated onto a layer of 0.6% agar (4 mL); and 4 mL of 0.6% agar was plated as the top layer. After culture for 21 days, colonies were counted.

### 3D spheroid formation

Spheroid formation of NIH3T3 cells expressing dynAP isoforms was examined as described previously [9]. Briefly, cells (500 cells/100 μL cell growth medium/well) were seeded on a PrimeSurface 96U U‐bottom 96‐well Ultralow Cell Adhesion Plate (Sumitomo Bakelite Co., Ltd., Tokyo, Japan) and cultured for 14 days. The well shape promoted the formation of single, centrally located spheroids. Spheroid formation was quantified by measuring the spheroid area with a Cell3iMager instrument (SCREEN Holdings Co., Ltd., Kyoto, Japan).

### Statistical analysis

Data were analyzed by Student's *t*‐test with Welch's correction, and *P* < 0.05 was considered statistically significant.

## Results and Discussion

### DynAP splicing variants

Figure 1 depicts exon–intron relationships in primary transcripts of the three dynAP splicing variants and delineates the encoded proteins predicted from the database sequences. Amino acid sequences of the three isoforms are also shown in Fig. S1.

### Expression of dynAP mRNAs in human cancer cell lines

**Fig. 2:**
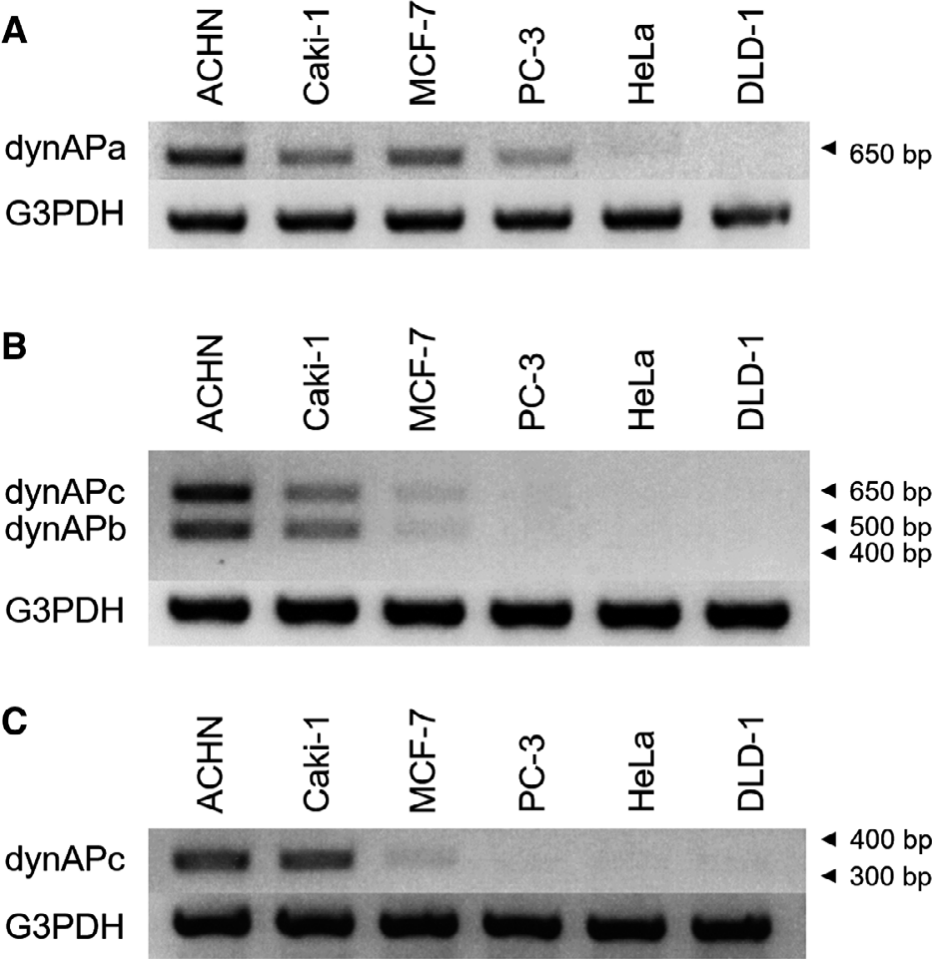
PCR products derived from dynAP variant mRNAs. (A) Primer set #1–2 produces a 649 bp DNA from dynAPa mRNA. (B) Primer set #0–2 produces a 649 bp DNA from dynAPc and a 497 bp DNA from dynAPb. (C) Primer set #0–10 produces a 319 bp DNA from dynAPc.

Primer set #1–2 produced a PCR product of 650 bp in ACHN, Caki‐1, MCF‐7, and PC‐3 human cancer cell lines, but not in HeLa or DLD‐1 cells (Fig. 2A). This size is in good agreement with that expected from the dynAPa mRNA. When primer set #0–2 was used, two products of ~ 500 and ~ 650 bp were abundant in ACHN and Caki‐1 cells, but present at a low level in MCF‐7 cells (Fig. 2B). The 500 bp product coincided with the product expected for dynAPb, while the 650 bp product may be derived from dynAPc, since this primer set should not produce any fragment from dynAPa. Next, we used primer set #0–10 to detect the dynAPc variant alone and observed a 319 bp product as expected (Fig. 2C). We confirmed that all three mRNAs were present in ACHN cells by cloning each cDNA and sequencing. Thus, mRNAs of all three variants were expressed in ACHN and Caki‐1 cells, and some other cell lines.

### Overexpression of dynAPa–c in NIH3T3 cells and subcellular localization

**Fig. 3:**
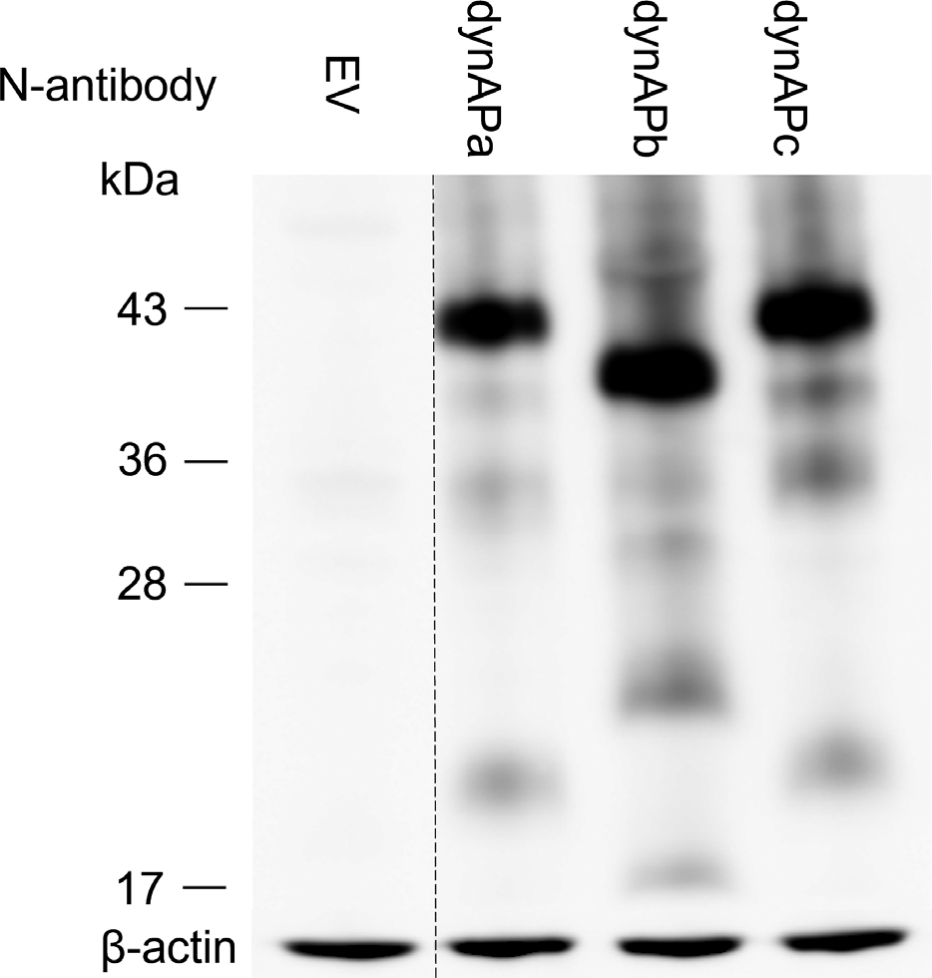
Overexpression of dynAP isoforms in NIH3T3 cells. DynAP proteins were detected by western blotting using N‐antibody. EV indicates empty vector. Lane 1, cells transfected with the empty vector (NIH3T3EV) as a negative control; Lane 2, dynAPa; Lane 3, dynAPb; Lane 4, dynAPc. β‐Actin was used as a loading control. The dashed line indicates two lanes that were spliced together.

The three dynAP variants were overexpressed in NIH3T3 cells, and dynAP proteins were detected by western blotting using N‐antibody (Fig. 3). This antibody can detect overexpressed dynAP, but not the endogenous one which is less expressed in the cells [9]. DynAPa and c migrated with a size of ~ 43 kDa in SDS/PAGE, and dynAPb migrated with a size of ~ 40 kDa. The sizes calculated from migration in SDS/PAGE are much larger than the molecular masses based on their amino acid sequences (22.5 kDa for dynAPa, 17.1 kDa for dynAPb, and 23 kDa for dynAPc). In unpublished work, we found that the anomalous migration of dynAPa on SDS/PAGE is due to its C‐terminal T/S‐rich region (X. Yin, T. Konishi, K. Horikawa, R. Tanaka, Y. Togo, T. Noda, M. Hosoi, M. Tsuchida, T. Kunoh, S. Wada, T. Nakamura, E. Tsuda, R. Sasaki, T. Mizukami & M. Hasegawa, unpublished data). This was confirmed by showing that a deletion mutant lacking the T/S‐rich region migrated to a position corresponding to its theoretical molecular weight. The amino acid sequence in this region of both dynAPb and dynAPc is the same as that in dynAPa (Fig. 1). Post‐translational modification in the T/S‐rich region, which is most likely *o*‐glycosylation, may cause this anomalous migration.

**Fig. 4:**
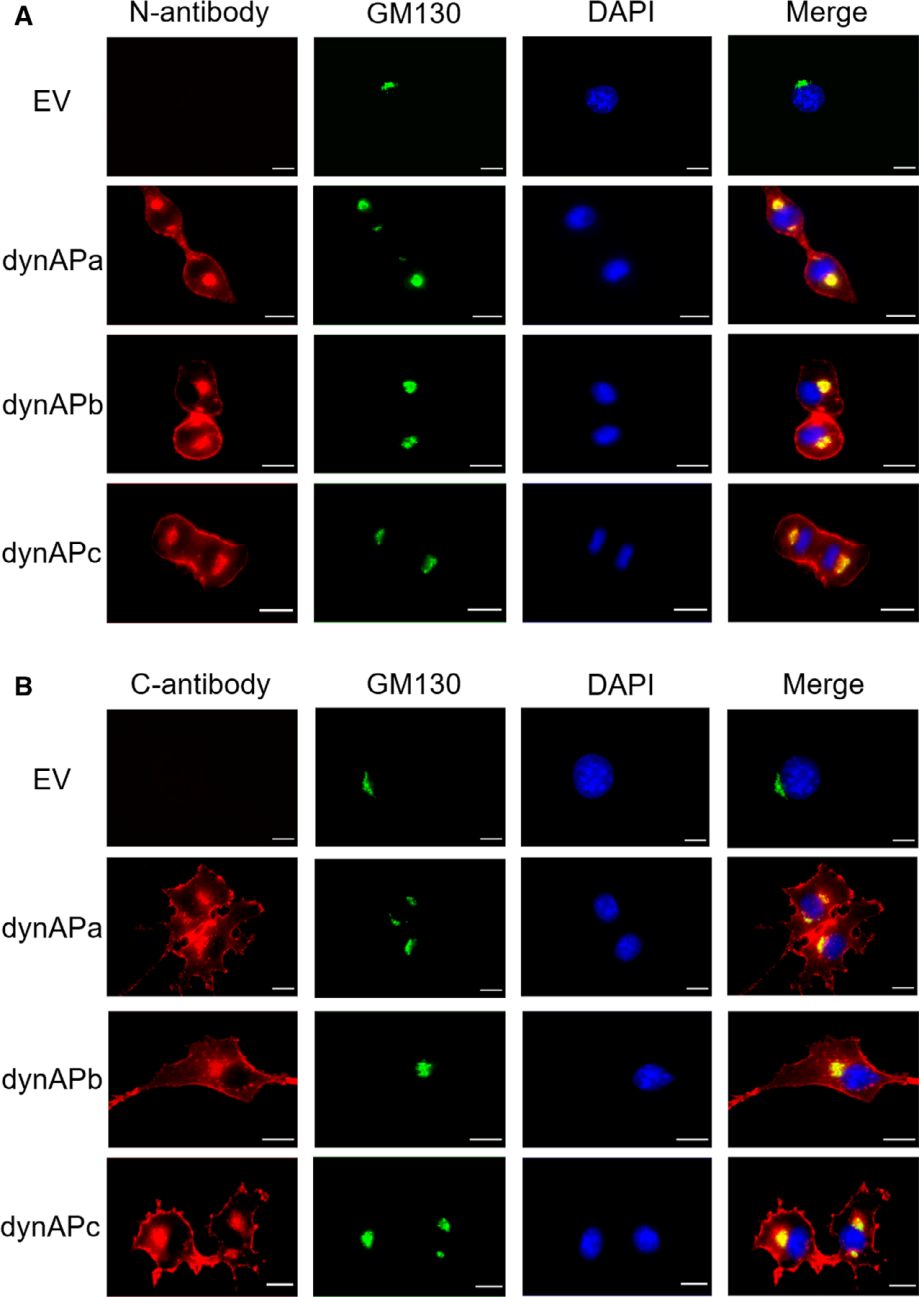
Subcellular localization of dynAP isoforms in NIH3T3 cells. (A) DynAP proteins detected using N‐antibody. (B) DynAP proteins detected using C‐antibody. EV indicates empty vector. Cells were cultured and stained as described in Materials and methods. Red indicates dynAP isoforms, green indicates Golgi marker GM130, and blue indicates DNA stained with DAPI. All isoforms were localized to the plasma membrane and Golgi apparatus. Size bars indicate 10 μm.

Next, we examined the subcellular localization of dynAP isoforms in NIH3T3 cells using N‐ and C‐antibodies (Fig. 4). The line scanning profiles of fluorescence intensity for each dynAP isoform and GM130 supported the idea that all isoforms were localized to the plasma membrane and Golgi apparatus (Fig. S2), consistent with the results obtained from HeLa cells overexpressing GFPdynAPa [6]. This result was confirmed with the KMST‐6 human cell line (Fig. S3). Flow cytometric analyses using C‐antibody showed that the C‐terminal regions of all isoforms are exposed to the outside of the cells (Fig. S4).

### Cell transformation

**Fig. 5:**
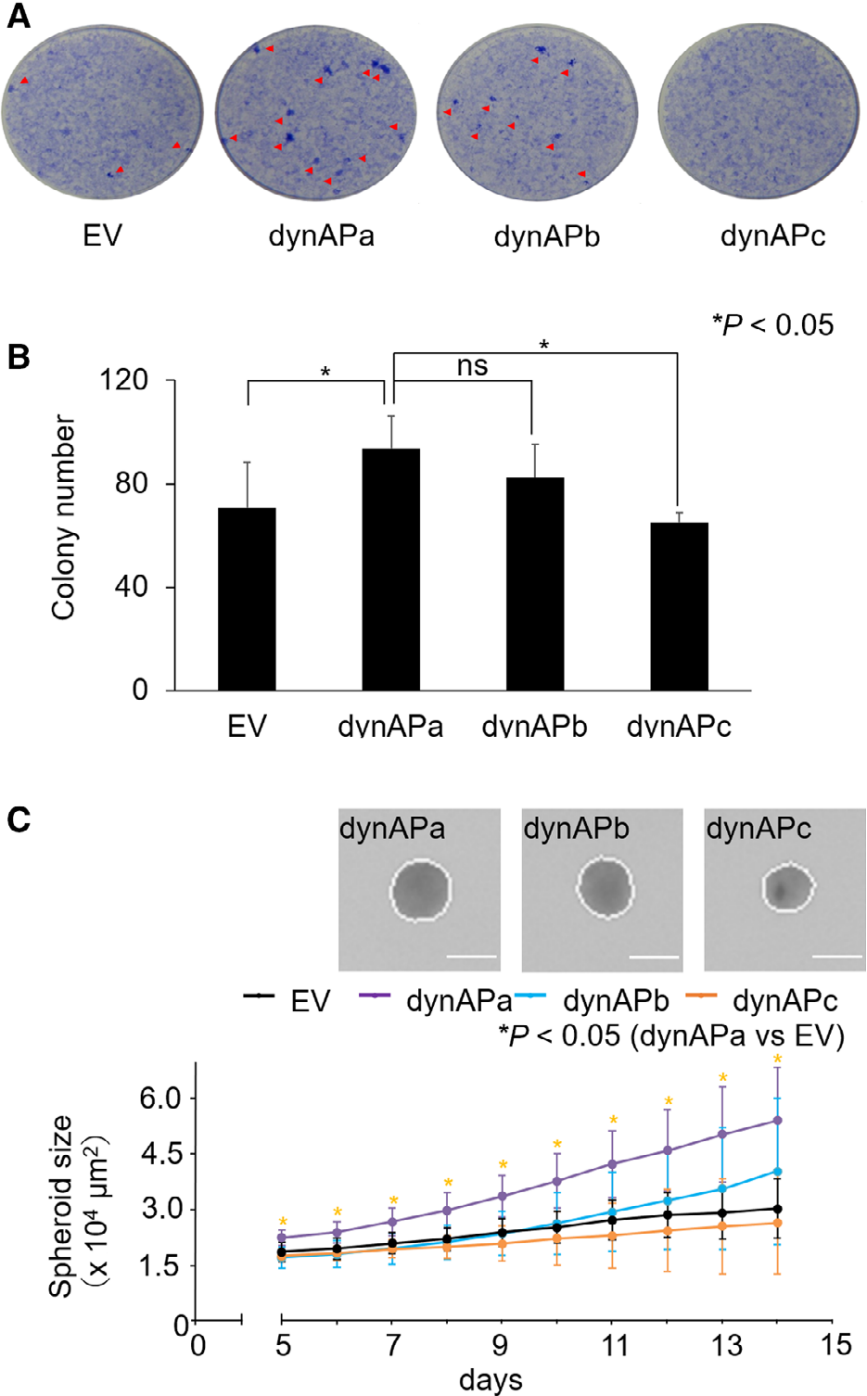
Cell transformation capability of dynAP isoforms. (A) Formation of foci. NIH3T3 cells expressing dynAP isoforms (10^4^ cells·2 mL^−1^ per well in 6‐well plates) were cultured for 15 days, fixed in ice‐cold methanol, and stained with 0.5% Crystal Violet to visualize colonies. EV indicates empty vector. Foci with diameters larger than 1 mm are indicated by red arrowheads. (B) Colony formation on soft agar. NIH3T3 cells expressing dynAP isoforms were cultured on soft agar for 21 days, and colonies with diameters larger than 100 mm were counted. EV indicates empty vector. Error bars show means ± standard deviation (SD; *n* = 4). (C) Spheroid formation. The spheroid‐forming ability of NIH3T3 cells expressing dynAP isoforms was investigated. The culture time‐dependent increase in spheroid area is shown (5–14 days). Asterisks indicate statistically significance (*P* < 0.05) by two sided *t*‐test. The ns means not statistically significant. Error bars show means ± SD (*n* = 8). (inset) Spheroid formation images acquired on the 14th day are shown. Size bars indicate 200 μm.

The formation of foci in 2D culture, colonies on soft agar, and spheroids in 3D culture is *in vitro* hallmarks of cell transformation [15]. We examined the *in vitro* cell transformation ability of dynAP isoforms. NIH3T3dynAPa cells produced many foci that were derived from loss of contact inhibition of cell proliferation; NIH3T3dynAPb cells produced fewer foci; and very few foci were produced by NIH3T3dynAPc cells and control NIH3T3 cells transformed with empty vector (Fig. 5A).

Colonies on soft agar with a diameter larger than 100 μm were counted, and NIH3T3dynAPa cells formed more colonies than control cells treated with empty vector (Fig. 5B). NIH3T3dynAPb cells tended to form more colonies than controls but were less robust than NIH3T3dynAPa cells. NIH3T3dynAPc cells behaved similarly to controls.

Measurement of spheroid area showed robust spheroid growth of NIH3T3dynAPa cells in a time‐dependent manner (Fig. 5C). The growth of spheroid by NIH3T3dynAPb cells was faster than that of control cells but considerably slower than that of NIH3T3dynAPa cells. NIH3T3dynAPc spheroids grew at a minimal rate similar to that of control cells. Thus, dynAPa displayed the highest cell transformation capability among the three isoforms. As shown in Fig. S1, dynAPa and dynAPc differ only at a few amino acids at the N terminus. The molecular mechanism by which this subtle difference affects the transformation activities is unknown. It is of interest that dynAPc completely lost transformation activity based on all criteria investigated. DynAPc has the same amino acid sequence as dynAPb at its N terminus, but there are differences in the remaining 55 amino acid residues (Fig. S1). It remains to be determined whether, like dynAPa, dynAPc can interact with dynamitin, p150^Glued^, and monomeric CCTδ. Identifying the amino acid residues required for cell transformation activity remains a goal of future studies.

## Conflict of interest

The authors declare no conflict of interest.

## Author contributions

XY, RS, TM, and MH designed the study. XY, SY, HK, RT, YT, M Hosoi, and MT acquired the data. XY, TK, SW, TN, RS, TM, and MH analyzed and interpreted the data. XY, TK, RS, TM, and MH wrote the manuscript. All authors agreed to submit the manuscript.

## Supporting information


**Fig. S1.** Amino acid sequences of dynAP isoforms. The amino terminus of dynAPa is numbered as 1. The * symbol denotes the same amino acid in dynAPa, and the ‐ symbol indicates the absence of an amino acid in dynAPb. Differences in sequences around the N terminus are colored red. TM, transmembrane region. N‐ and C‐antigens indicate peptides used for raising N‐ and C‐antibodies, respectively.
**Fig. S2.** Line‐scan profiles of fluorescence intensity for dynAP isoforms (solid line) and Golgi marker GM130 (dot line) were generated from the data of Figure 4 (A) and (B). Size bars indicate 10 μm.
**Fig. S3.** Subcellular localization of dynAP isoforms in KMST‐6 cells. (A) DynAP isoforms were transiently expressed in KMST‐6 cells and detected with C‐antibody. (B) Line‐scan profiles of fluorescence intensity for dynAP isoforms (solid line) and Golgi marker GM130 (dot line) were generated from the data of (A). (C) N‐terminally Flag‐tagged dynAP isoforms were transiently expressed in KMST‐6 cells and detected with Flag‐antibody. EV indicates empty vector. Cells were cultured and stained as described in Materials and Methods. Red indicates dynAP isoforms, green indicates GM130, and blue indicates DNA stained with DAPI. (D) Line‐scan profiles of fluorescence intensity for dynAP isoforms (solid line) and Golgi marker GM130 (dot line) were generated from the data of (C). All isoforms were localized to the plasma membrane and Golgi apparatus. Size bars indicate 10 μm.
**Fig. S4.** Flow cytometric analysis demonstrating that the C‐terminal regions of dynAP isoforms are exposed to the outside of the cells. DynAPa, b, and c were separately expressed in NIH3T3 cells using retroviral vectors. Populations of cells binding to the C‐antibody and expressing enhanced green fluorescent protein (EGFP) were analyzed by fluorescence‐activated cell sorting (FACS). EV indicates empty vector (pMY‐IRES‐EGFP). IgG indicates nonspecific IgG used as a control for antibody binding. The percentage of cells binding to the C‐antibody and expressing EGFP were 94.31% for dynAPa, 90.94% for dynAPb, 91.81% for dynAPc, and 1.44% for empty vector controls.

## Data Availability

The original data are available upon reasonable request.
