# Supplementary material for: Expression and cell transformation activity of dynactin‐associated protein isoforms
Source: FEBS Open Bio. 2021 Jul 16;11(8):2110–7. doi: 10.1002/2211-5463.13202 (PMC8329785; doi:10.1002/2211-5463.13202)
Supplement: Supplementary file 1 — Fig. S1. Amino acid sequences of dynAP isoforms. The amino terminus of dynAPa is numbered as 1. The * symbol denotes the same amino acid in dynAPa, and the ‐ symbol indicates the absence of an amino acid in dynAPb. Differences in sequences around the N terminus are colored red. TM, transmembrane region. N‐ and C‐antigens indicate peptides used for raising N‐ and C‐antibodies, respectively. Fig. S2. Line‐scan profiles of fluorescence intensity for dynAP isoforms (solid line) and Golgi marker GM130 (dot line) were generated from the data of Figure 4 (A) and (B). Size bars indicate 10 μm. Fig. S3. Subcellular localization of dynAP isoforms in KMST‐6 cells. (A) DynAP isoforms were transiently expressed in KMST‐6 cells and detected with C‐antibody. (B) Line‐scan profiles of fluorescence intensity for dynAP isoforms (solid line) and Golgi marker GM130 (dot line) were generated from the data of (A). (C) N‐terminally Flag‐tagged dynAP isoforms were transiently expressed in KMST‐6 cells and detected with Flag‐antibody. EV indicates empty vector. Cells were cultured and stained as described in Materials and Methods. Red indicates dynAP isoforms, green indicates GM130, and blue indicates DNA stained with DAPI. (D) Line‐scan profiles of fluorescence intensity for dynAP isoforms (solid line) and Golgi marker GM130 (dot line) were generated from the data of (C). All isoforms were localized to the plasma membrane and Golgi apparatus. Size bars indicate 10 μm. Fig. S4. Flow cytometric analysis demonstrating that the C‐terminal regions of dynAP isoforms are exposed to the outside of the cells. DynAPa, b, and c were separately expressed in NIH3T3 cells using retroviral vectors. Populations of cells binding to the C‐antibody and expressing enhanced green fluorescent protein (EGFP) were analyzed by fluorescence‐activated cell sorting (FACS). EV indicates empty vector (pMY‐IRES‐EGFP). IgG indicates nonspecific IgG used as a control for antibody binding. The percentage of cells binding t [file FEB4-11-2110-s001.pdf]

```
              1       10      20      30      40      50
dynAPa     MVADI KGNEQ IEKYS WREAC DTGSS RMDRK HGKYI LNVEH SENQP PITHP NDQEA
dynAPb    MEYQLL** ***** ***** ***** ***** ***** ***** ***** ***** ----- -----
dynAPc    MEYQLL** ***** ***** ***** ***** ***** ***** ***** ***** ***** *****
                              N-antigen (20-33)

              60      70      80      90      100     110
           HSSIC WCLPS NDITS DVSPN LTGVC VNPGI LAHSR CLQSE SCNTQ VKEYC RNDWS
           ----- ----- ----- ----- ----- ----- ----- ----- ----- ***** *****
           ***** ***** ***** ***** ***** ***** ***** ***** ***** ***** *****

                 120     130     140     150     160
           MWKVF LACLL ACVIM TAIGV LIICL VNNKG SANSS IVIQL STNDG ECVTV KPGTP
           ***** ***** ***** ***** ***** ***** ***** ***** ***** ***** *****
           ***** ***** ***** ***** ***** ***** ***** ***** ***** ***** *****
                          TM

              170     180     190     200     210
           SPACP PTMTT TSTVP ASTAT ESTTS TATAA TTSTE PITVA PTDHL
           ***** ***** ***** ***** ***** ***** ***** ***** *****
           ***** ***** ***** ***** ***** ***** ***** ***** *****
                                      C-antigen (194-210)
```

Supplementary Fig. 1. Amino acid sequences of dynAP isoforms. The amino terminus of dynAPa is numbered as 1. The * symbol denotes the same amino acid in dynAPa, and the - symbol indicates the absence of an amino acid in dynAPb. Differences in sequences around the N-terminus are colored red. TM, transmembrane region. N- and C-antigens indicate peptides used for raising N- and C-antibodies, respectively.

(A)

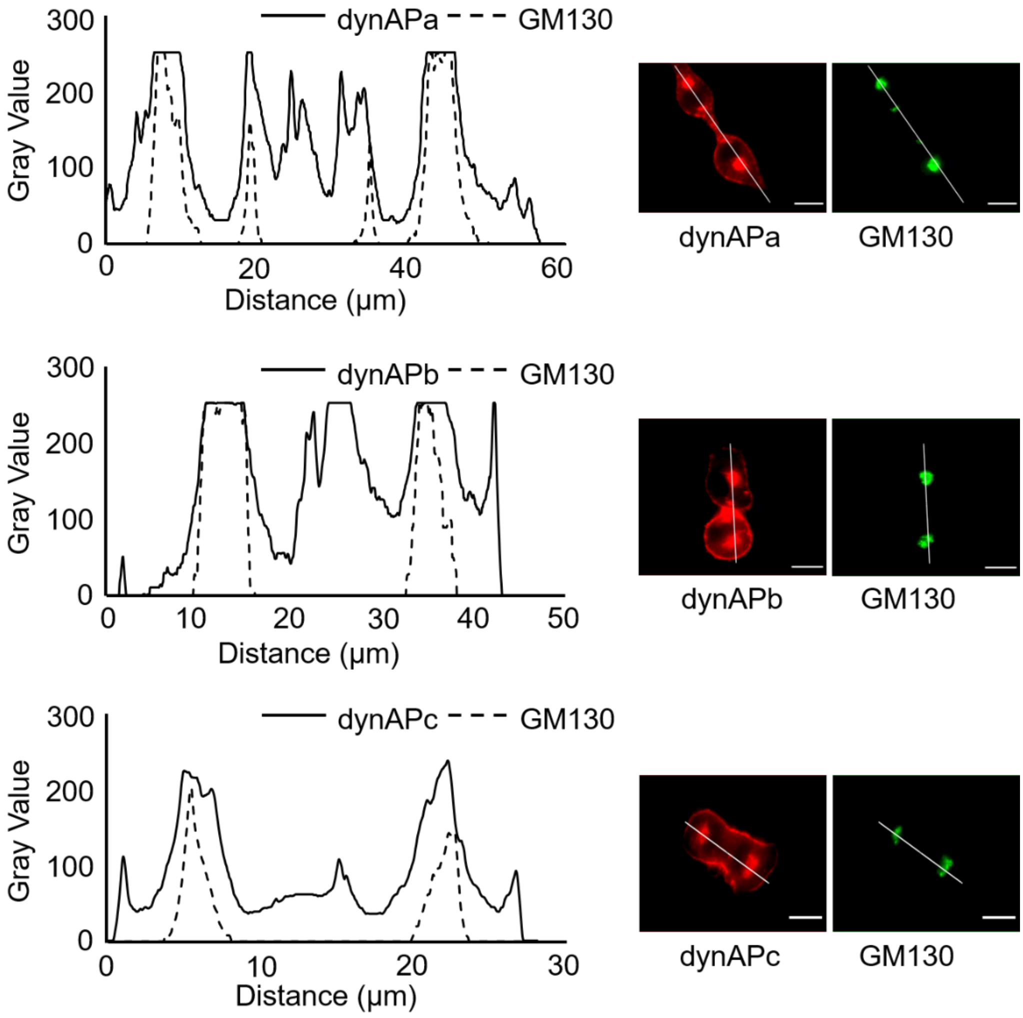

dynAPa
GM130
Gray Value
Distance (μm)
dynAPa
GM130
dynAPb
GM130
Gray Value
Distance (μm)
dynAPb
GM130
dynAPc
GM130
Gray Value
Distance (μm)
dynAPc
GM130

(B)

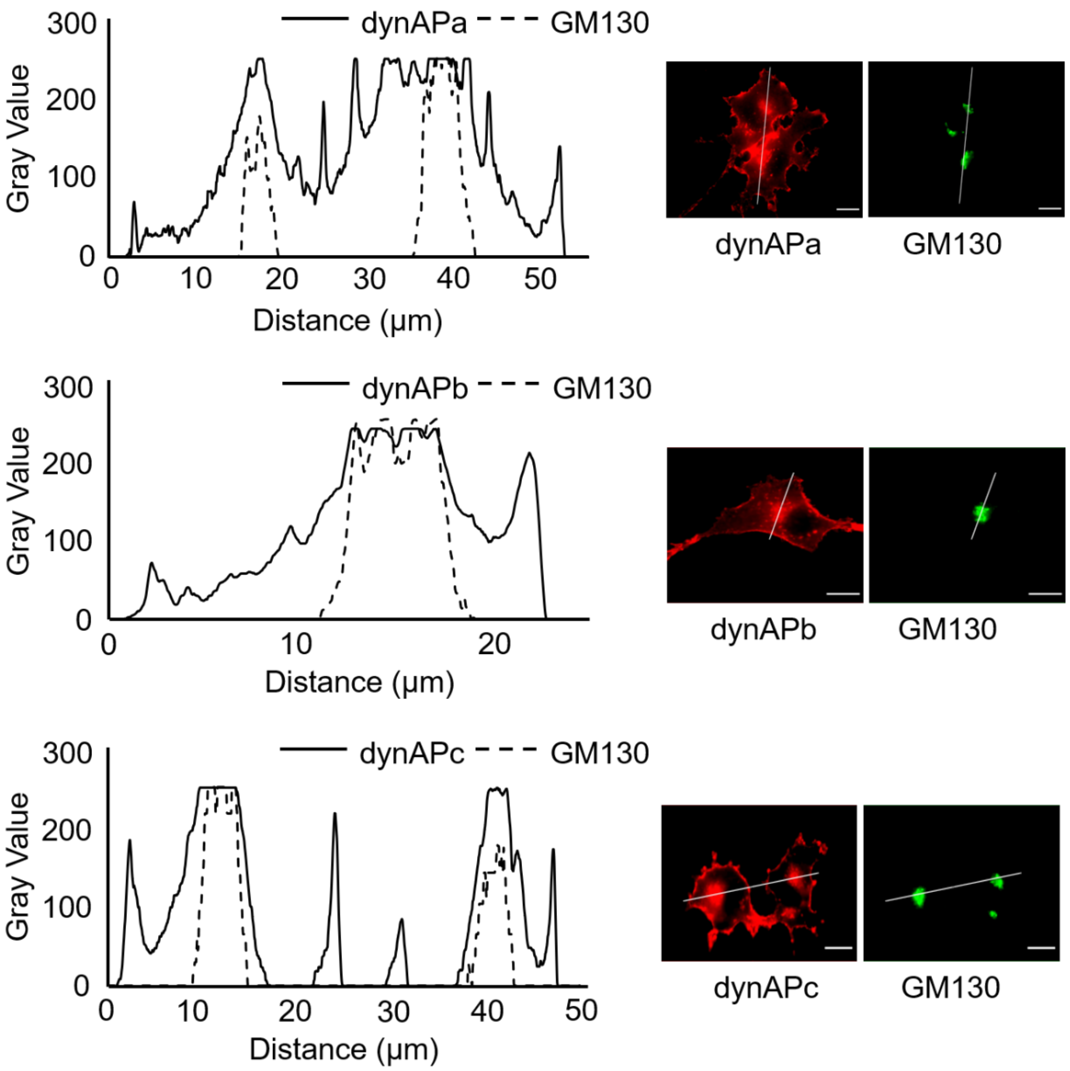

Supplementary Fig. 2. Line-scan profiles of fluorescence intensity for dynAP isofoms (solid line) and Golgi marker GM130 (dot line) were generated from the data of Fig. 4 (A) and (B). Size bars indicate 10 µm.

(A)

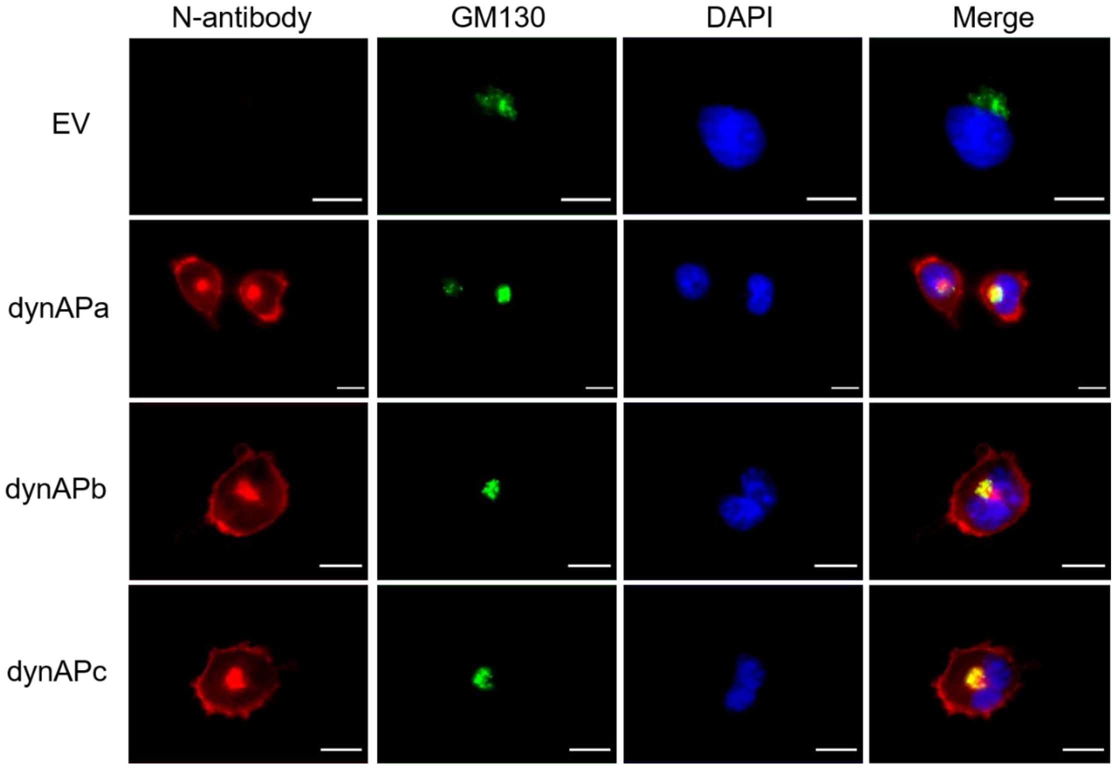

(B)

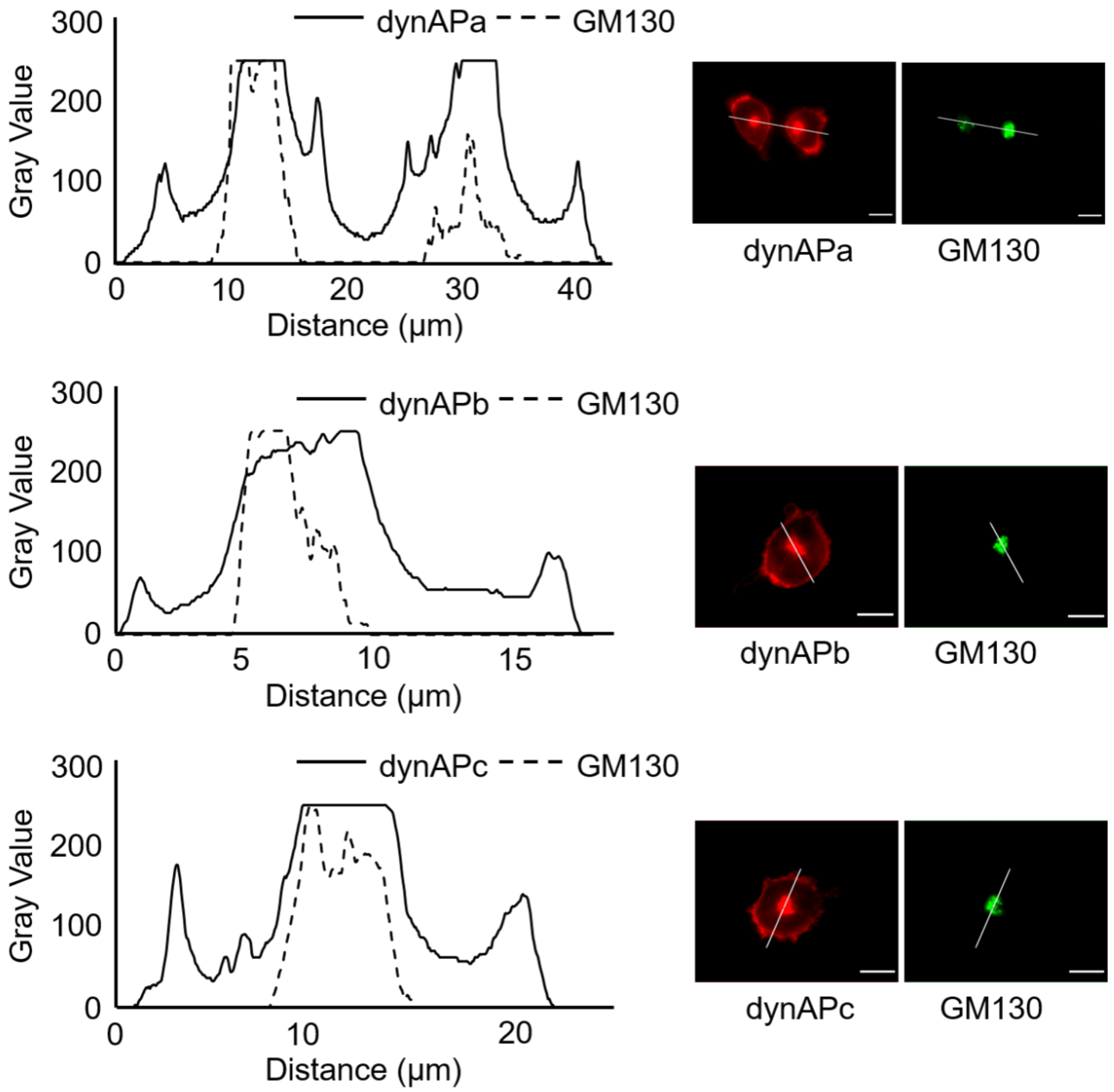
dynAPa
GM130
300
200
100
0
Gray Value
0
10
20
30
40
Distance (μm)
dynAPa
GM130
dynAPb
GM130
300
200
100
0
Gray Value
0
5
10
15
Distance (μm)
dynAPb
GM130
dynAPc
GM130
300
200
100
0
Gray Value
0
10
20
Distance (μm)
dynAPc
GM130

(C)

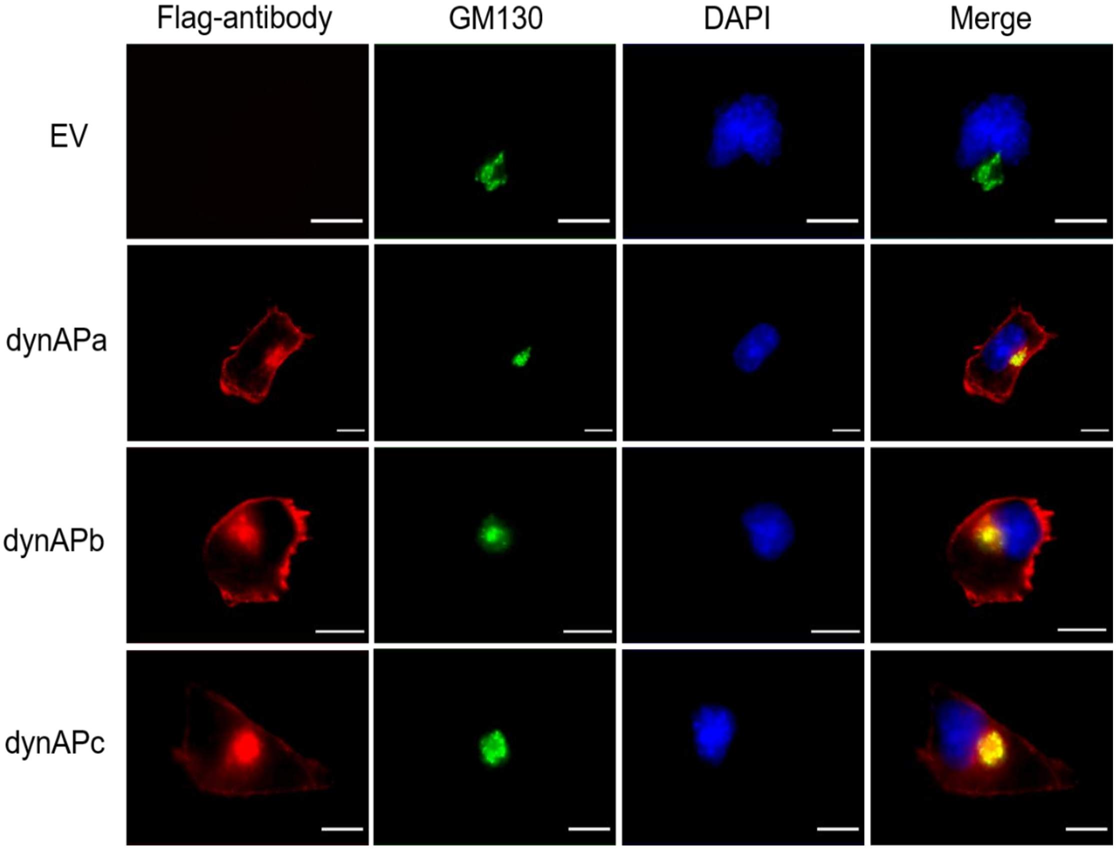

(D)

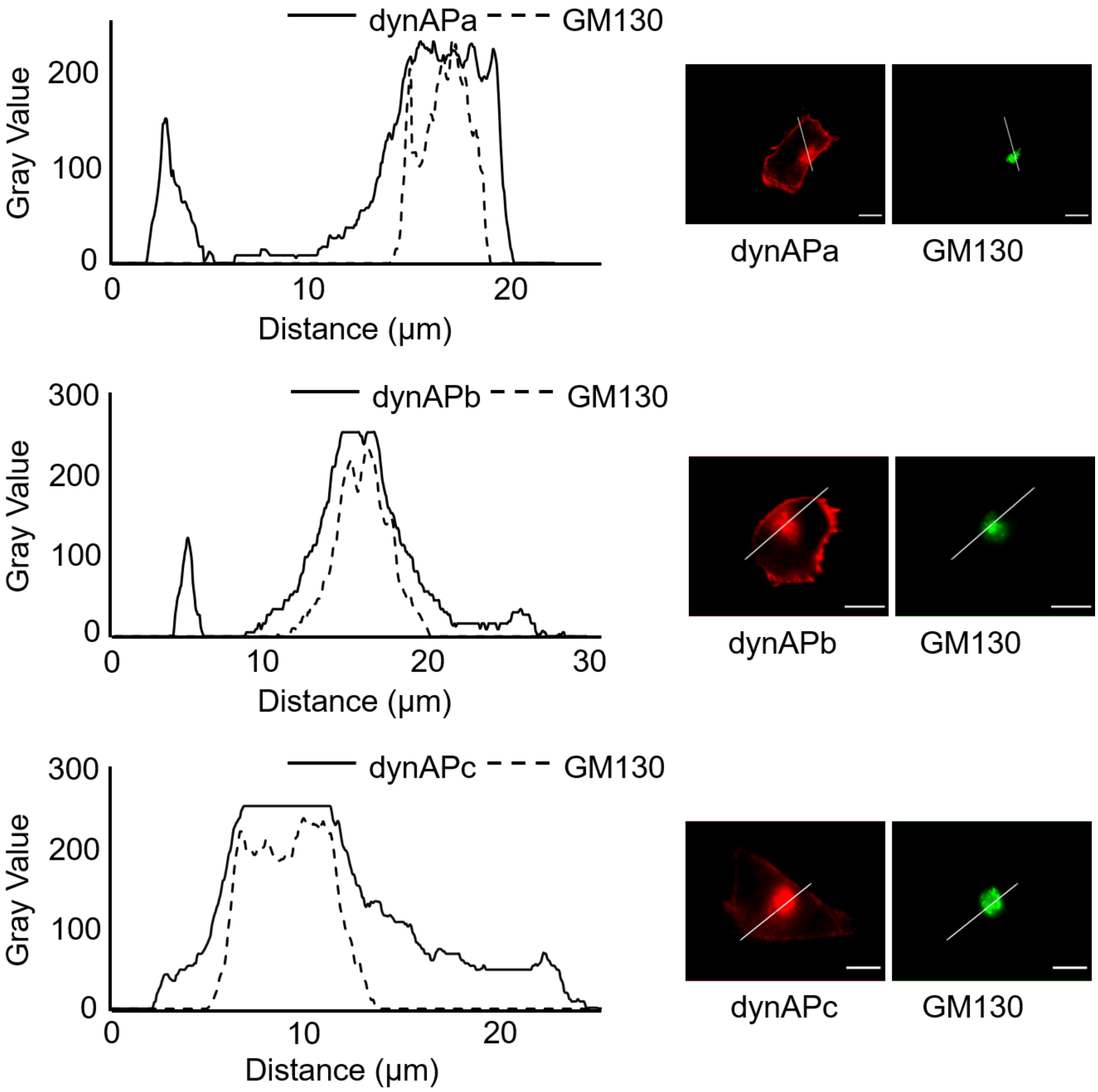
dynAPa
GM130
Gray Value
200
100
0
0
10
20
Distance (μm)
dynAPa
GM130
dynAPb
GM130
Gray Value
300
200
100
0
0
10
20
30
Distance (μm)
dynAPb
GM130
dynAPc
GM130
Gray Value
300
200
100
0
0
10
20
Distance (μm)
dynAPc
GM130

Supplementary Fig. 3. Subcellular localization of dynAP isoforms in KMST-6 cells. (A) DynAP isoforms were transiently expressed in KMST-6 cells and detected with C-antibody. (B) Line-scan profiles of fluorescence intensity for dynAP isofoms (solid line) and Golgi marker GM130 (dot line) were generated from the data of (A). (C) N-terminally Flag-tagged dynAP isoforms were transiently expressed in KMST-6 cells and detected with Flag-antibody. EV indicates empty vector. Cells were cultured and stained as described in Materials and Methods. Red indicates dynAP isoforms, green indicates GM130, and blue indicates DNA stained with DAPI. (D) Line-scan profiles of fluorescence intensity for dynAP isofoms (solid line) and Golgi marker GM130 (dot line) were generated from the data of (C). All isoforms were localized to the plasma membrane and Golgi apparatus. Size bars indicate 10 μm.

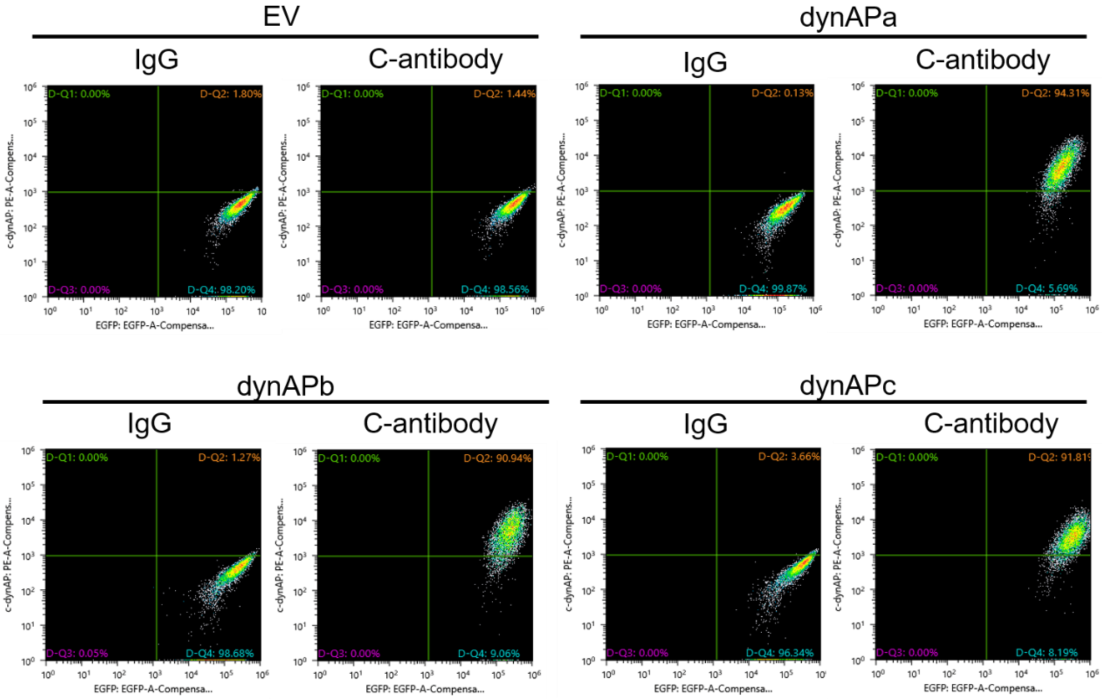

Supplementary Fig. 4. Flow cytometric analysis demonstrating that the C-terminal regions of dynAP isoforms are exposed to the outside of the cells. DynAPa, b, and c were separately expressed in NIH3T3 cells using retroviral vectors. Populations of cells binding to the C-antibody and expressing enhanced green fluorescent protein (EGFP) were analyzed by fluorescence-activated cell sorting (FACS). EV indicates empty vector (pMY-IRES-EGFP). IgG indicates nonspecific IgG used as a control for antibody binding. The percentage of cells binding to the C-antibody and expressing EGFP were 94.31% for dynAPa, 90.94% for dynAPb, 91.81% for dynAPc, and 1.44% for empty vector controls.
